# Guanosine modulates K^+^ membrane currents in SH-SY5Y cells: involvement of adenosine receptors

**DOI:** 10.1007/s00424-022-02741-4

**Published:** 2022-09-01

**Authors:** Giuditta Gambino, Giuseppe Giglia, Daniele Gallo, Miriana Scordino, Costanza Giardina, Mariachiara Zuccarini, Patrizia Di Iorio, Patricia Giuliani, Francisco Ciruela, Giuseppe Ferraro, Giuseppa Mudò, Pierangelo Sardo, Valentina Di Liberto

**Affiliations:** 1 Department of Biomedicine, Neuroscience and Advanced Diagnostic, University of Palermo, Palermo, Italy; 2 Department of Medical, Oral and Biotechnological Sciences, “G. D’Annunzio” University of Chieti-Pescara, Chieti, Italy; 3 Center for Advanced Studies and Technology, CAST, “G. D’Annunzio” University Foundation, Chieti, Italy; 4 Department of Pathology and Experimental Therapeutics, University of Barcelona, L’Hospitalet de Llobregat, Barcelona, Spain; 5 Neuropharmacology and Pain Group, Neuroscience Program, Institut d’Investigació Biomèdica de Bellvitge, IDIBELL, L’Hospitalet de Llobregat, Barcelona, Spain

**Keywords:** Purines, Potassium channels, Patch-clamp, SH-SY5Y cells, Electrophysiology, Cellular excitability

## Abstract

Guanosine (GUO), widely considered a key signaling mediator, is implicated in the regulation of several cellular processes. While its interaction with neural membranes has been described, GUO still is an orphan neuromodulator. It has been postulated that GUO may eventually interact with potassium channels and adenosine (ADO) receptors (ARs), both particularly important for the control of cellular excitability. Accordingly, here, we investigated the effects of GUO on the bioelectric activity of human neuroblastoma SH-SY5Y cells by whole-cell patch-clamp recordings. We first explored the contribution of voltage-dependent K^+^ channels and, besides this, the role of ARs in the regulation of GUO-dependent cellular electrophysiology. Our data support that GUO is able to specifically modulate K^+^-dependent outward currents over cell membranes. Importantly, administering ADO along with GUO potentiates its effects. Overall, these results suggested that K^+^ outward membrane channels may be targeted by GUO with an implication of  ADO receptors in SH-SY5Y cells, but also support the hypothesis of a functional interaction of the two ligands. The present research runs through the leitmotif of the deorphanization of GUO, adding insight on the interplay with adenosinergic signaling and suggesting GUO as a powerful modulator of SH-SY5Y excitability.

**Supplementary Information:**

The online version contains supplementary material available at 10.1007/s00424-022-02741-4.

## Introduction

Guanine-based purines (GBPs), including guanosine (GUO), are important intracellular metabolites and extracellular purinergic signaling molecules mediating several effects within the central nervous system (CNS) [1, 2].

More generally, GUO is released in the brain upon both physiological and pathological conditions [3]. Similarly to Adenosine (ADO), GUO is released as such by cells, although both nucleosides largely derive from the activity of ecto- and released 5′-nucleotidase [4]. In turn, these nucleosides are metabolized into the corresponding bases by the extracellular Purine Nucleoside Phosphorylase [5, 6], thus generating a physiological equilibrium in the amounts and activities of extracellular nucleosides.

Undoubtedly, GUO binding sites that correspond to putative G protein-coupled receptors (GPCRs) at rat brain membranes have already been described, together with the related downstream intracellular pathways [7–9]. However, since GUO receptors have not been identified and/or characterized yet, GUO still is an orphan neuromodulator [1].

Interestingly, several GUO effects involve the participation of ADO receptors (ARs), especially A_1_R and/or A_2A_R, which belong to the P1 family [7, 10]. These two GPCRs are highly expressed in the brain, show a high affinity for ADO, and exert both presynaptic and postsynaptic neuromodulatory effects [11, 12]. In the synaptic context, ARs functional interplay is mediated by A_1_R–A_2A_R heteromerization at glutamatergic terminals [13, 14], and heterodimerization with other GPCRs [15, 16], thus leading to integrated mechanisms of neuromodulation and regulation of both physiological and pathological processes.

Since some neuromodulatory effects of GUO are precluded by selective AR agonists/antagonists [7, 17], a functional interplay between these purines has been proposed [1]. In detail, it has been recently assumed that (i) GUO may also bind to ARs, triggering different effects to those promoted by ADO; (ii) specific GUO receptors share some features with ARs and bind both ligands, likely with different affinities; and (iii) GUO binding sites generate an allosteric modulation of ARs, dependently on heteroreceptors complexes [18]. Importantly, it has been recently demonstrated that ADO is able to displace [^3^H]GUO binding with a potency order similar to GUO [7] and that GUO-induced effects require both A_1_R and A_2A_R co-expression. It was indeed evidenced that GUO may act as a negative allosteric modulator of A_2A_R only when A_1_R is present [10, 19].

Independently of its adenosinergic effect, GUO has been suggested to act as a multitarget signaling molecule. For instance, GUO interferes with glutamatergic neurotransmission, both at the transporter and receptor level [20, 21]. In addition to this, GUO-dependent neuroprotection has been associated with calcium-activated potassium channel modulation in SH-SY5Y cells [22]. Also, GUO treatment increases the expression of inward rectifier K^+^ channels in cultured rat cortical astrocytes [23], suggesting a regulation of potassium currents, which may influence cell excitability and neurotransmitter release/uptake in both neuronal and glial cells.

The present research focuses on the cellular bioelectric activity of SH-SY5Y cells that have always been considered an invaluable experimental model for studying the effects of GUO in the attempt to fully uncover its role within purinergic signaling [22, 24, 25]. Indeed, this cell line if adequately stimulated elicited voltage membrane currents constituted of a fast transient inward Na^+^ and sustained outward K^+^ currents [26–29]. In this light, we aimed to assess the putative influence of GUO on the electrophysiological activity of human neuroblastoma SH-SY5Y cells by whole-cell patch-clamp experiments, exploring the contribution of voltage-dependent K^+^ currents in the modulation of cellular excitability. Indeed, despite the large body of evidence demonstrating the ability of GUO to regulate neuronal physiology, GUO-mediated effects on discrete membrane properties in SH-SY5Y cells remain enigmatic.

## Materials and methods

### Cell cultures and treatment

The neuronal-like cell line SH-SY5Y was cultured in T25 tissue culture flasks as described in Nuzzo et al. 2021 [30]. Briefly, cells were grown in complete Dulbecco’s Modified Eagle’s Medium and F12 (DMEM/F12; 1:1), supplemented with 10% fetal bovine serum (FBS), 100 U/mL penicillin, and 100 U/mL streptomycin and 2 mM l-glutamine, in a humidified atmosphere of 95% air and 5% CO_2_ at 37 °C. The cell culture medium was replaced every three days, and the cells were sub-cultured once they reached 90% confluence, usually once a week. For electrophysiological recordings, cells were plated in cell culture dishes 35 mm at a density of 4 × 10^5^ cells/dish, usually 48 h before patch-clamp recordings. For acute assessment of the effect of treatments (Experiment 1), the cell medium was removed on the day of whole-cell recordings and substituted with an extracellular bath solution composed of (in mM): 125 NaCl; 4 KCl; 2 CaCl2; 1 MgCl2; 10 HEPES; 10 glucose (pH adjusted to 7.4 with NaOH) and tetrodotoxin (TTX, 1 µM), to eliminate the contribution of the voltage-dependent Na^+^ channels. Then, GUO (100 µM), ADO (100 µM), the A1 adenosine receptor antagonist/inverse agonist (1,3-dipropyl-8-cyclopentylxanthine—DPCPX, 1 µM), the A2A receptor antagonist/inverse agonist [4-(2-[7-amino-2-{2-furyl}{1,2,4}triazolo{2,3a}{1,3,5}triazin-5ylamino]ethylphenol]-ZM241385, 1 µM), and the unselective blocker tetraethylammonium (TEA, 20 mM) were dissolved in the extracellular bath solution 15 min after TTX at final dosages previously reported for modulating purinergic signaling [21, 22, 25, 31]. Experimental groups obtained following drug administration are herein enlisted: CTR, GUO, ADO, ZM241385, DPCPX, DPCPX + GUO, ZM241385 + GUO, DPCPX + ADO, ZM241385 + ADO, and lastly GUO + ADO. The co-administration of AR antagonists/inverse agonists with GUO or ADO was performed dissolving in the extracellular solution first the antagonist and then GUO or ADO, whereas in the GUO + ADO group, both drugs were concurrently dissolved. A further experimental group was obtained when GUO was administered after TEA in the extracellular bath solution in order to obtain additional confirmation that the recorded outward currents are predominantly K^+^ mediated (i.e., GUO + TEA group). Control cells received an equal amount of vehicle.

For assessment of more prolonged effects of GUO and ADO alone (experiment 2), cells were incubated with GUO and/or ADO (100 µM) for 24 h before electrophysiological recordings in their culture medium in which the extracellular concentration of K^+^ was 4 mM in order to maintain the proper ratio with internal K^+^, similarly to the extracellular solution used for acute experiments. To eliminate the contribution of the voltage-dependent Na^+^ channels, tetrodotoxin (TTX, 1 µM) was added to the culture medium 15 min before the experimental session for recordings of voltage-dependent currents.

Each experimental group contained cells recorded from at least three different experiments performed in independent cultured preparation.

All drugs and toxins were purchased from Merck-Sigma-Aldrich (Merk Life Science S.r.l., Mi, Italy).

### Electrophysiological recordings

Recording electrodes were prepared from borosilicate glass capillaries (1.5 mm of outer diameter, 0.86 mm of inner diameter, furnished by Sutter Electrical Instruments), pulled by a PC-10 Narishige International vertical puller in order to obtain a pipette resistance of 2–5 MΩ. They were filled with an internal solution having the following compositions: KCl (140 mM), HEPES (10 mM), NaCl (4 mM), EGTA (0,8 mM), and MgCl2 (2 mM) (pH = 7.2 adjusted with KOH), to test the possible influence of K^+^ ions on the response obtained and on the amplitude of outward currents. Patch-clamp recordings were carried out at room temperature (25 °C). The concentration of K^+^ ions in our experimental conditions was always *K*_out_ = [4 mM] and *K*_in_ = [141 mM], therefore its equilibrium potential (*E*_ion_) computed by the Nernst equation for a temperature of 25 °C is E(k) =  − 91,478 mV, as in Santillo et al. 2014 that indicated the ideal solution to focus on K^+^ currents. Gigaseal resistance ranged 5–20 GΩ. Once gigaseal formation was obtained, the fast capacitance was compensated and then whole-cell configuration was obtained by a gentle suction with a holding potential of − 90 mV, known for reducing the intrinsic variability of this cell line [27, 29]. Recordings were performed only when series resistance (*R*_s_) was less than twice the electrode resistance (*R*_s_ < 2*R*_e_) and, in any case, not greater than 15 MΩ, considered as inclusion/exclusion criteria for cells included in statistical analyses.

**Fig. 1:**
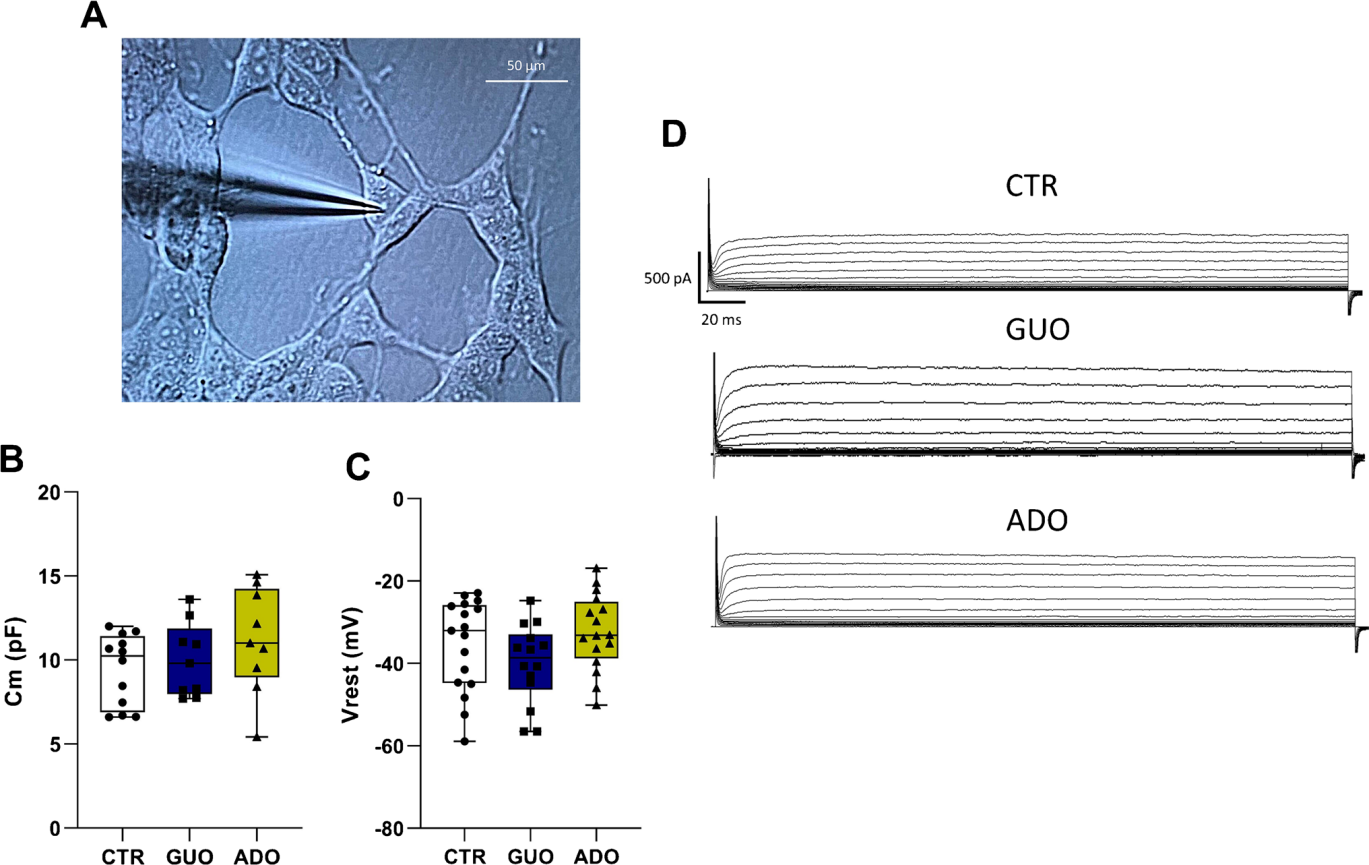
**A** Representative photomicrographs of SH-SY5Y cells during whole-cell patch recordings (magnification 40X). **B**, **C** Passive membrane properties. Box-and-whisker plots depict the minimum, 25th percentile, median, 75th percentile, and the maximum of *C*_m_ and *V*_rest_ in the experimental groups. Mean *C*_m  _values **(B)** and mean *V*_rest_ values in controls (CTR), in cells treated with guanosine (GUO) and adenosine (ADO) **(C)**. **D** Representative total membrane ionic currents recorded in the SH-SY5Y cells following different treatments. Outward currents in CTR, GUO and ADO were elicited holding the cell membrane at – 90 mV and stepping in 10-mV increments from − 90 to + 50 mV, bath solution contained TTX 1 µM

Morphologically, SH-SY5Y cells were identified by a 40 × immersive microscope with a round-to-oval soma and at least one or more branched processes (as represented in Fig. 1A). Immediately after the whole-cell configuration and before any further compensation, the passive membrane properties were measured, i.e., the membrane capacitance (*C*_m_) and the membrane voltage (*V*_m_). In particular, the *V*_m_ was recorded in current-clamp (C-clamp) mode at 0-pA holding level which is considered the most probable resting potential (*V*_rest_) in natural conditions [26, 27]. Furthermore, to guarantee the adequate space clamp for each recorded cell we monitored the time constant to be within the reported cut-off typical of single cells and not of SH-SY5Y aggregates (as already indicated by Sonnier [32]). Then, a stimulation protocol in voltage-clamp (V-clamp) mode allowed the evaluation of membrane currents for the assessment of the effects of all the drugs used. To generate total currents, cells were held at a holding level of − 90 mV and stepped by 15 subsequent depolarizing steps of 10-mV amplitude each lasting 275 ms, in a physiological range of potentials from − 120 to + 20 mV, with a prepulse at − 120 mV, and in a range from − 90 to + 50 mV, indicated for collection of outward currents. In standard ionic conditions, current–voltage “I-V” relationships of steady membrane currents were plotted and analyzed, considering the output current (pA) vs voltage steps applied (mV). Linear leak subtraction with the P/N method was used for voltage-clamp recordings to provide subtracted curves (as reported in I-V plots) and exclude the influence of leak currents on the active voltage-dependent currents.

Conductances (*G*) were calculated from the mean amplitudes of currents elicited by conditioning voltage steps using the equation: *G* = *I*/(*V*_m_ − *E*_k_) where I is the peak mean currents elicited during the conditioning depolarization *V*_m_ and *E*_k_ is the reversal potential for the K^+^ ion, as computed by Nernst equation. For the purpose of studying K^+^ activation curves, mean maximal conductance (*G*_max_) was used to normalize the mean conductivity values G, where *G*_max_ is the mean maximal conductance obtained by the mean of all maximum conductances resulting at the depolarizing voltage step of sufficient magnitude to elicit maximum K^+^ conductivity (i.e., + 50 mV). “*G*/*G*_max_” relationships of K^+^ activation curves were plotted and analyzed, as in [26].

In experiment 2, to test the ability of these cells to emit action potentials (AP), changes in membrane potential inducing AP in C-clamp mode were evoked by initially passing a hyperpolarizing current to a membrane potential value ranging from – 50 to – 110 mV, followed by 15 subsequent depolarizing current pulses of 500-ms duration in 10-pA steps, in an extracellular bath devoid of TTX. The electrophysiological parameters recorded were the mean amplitude of AP (“AP amplitude,” mV), the time to reach a positive peak (“depolarization time,” ms), the time to repolarize and reach the negative peak (“repolarization time,” ms), and the frequency of events, i.e., of the AP evoked per experimental group. Only cells emitting overshoot AP (over 0 mV) were analyzed for further comparison following a 24-h treatment with GUO or ADO versus CTR.

Electrical activity was recorded using a Multiclamp 700B amplifier (Axon Instruments, Molecular Devices, CA, USA, 300 Hz–3-kHz bandpass). Signals were filtered at 3 kHz and acquired at a 10-kHz sampling rate. The raw electrical activity was digitally converted; in addition to fully storing it on a computer for offline analysis, raw activity was passed through a software window discriminator and digital signals were online displayed. The electrophysiological procedures were performed following previous literature [26, 33–35]. All computer operations were performed using the pClamp package, version 10.5.0, for stimulus generation, data display, acquisition, and storage (Molecular Devices, Berthoud, CO, USA).

### Statistical analysis

Statistical analysis was performed by GraphPad Prism 9.02 (San Diego, CA, USA). Data were evaluated by a two-way ANOVA, followed by Bonferroni’s post hoc test for significant differences for within- and between-subject comparisons, considering the effect of “voltage,” “pharmacological treatment,” and their interaction. When analyzing cumulative mean values between groups of *C*_m_, *V*_rest_, and of the electrophysiological parameters recorded in C-clamp mode, a one-way ANOVA followed by Bonferroni’s post hoc test was applied. The analyses of *G*_max_ of GUO or ADO respectively vs CTR were performed by an unpaired *t*-test. All values are presented as the mean ± standard error of the mean. Data represented as box-and-whiskers plots depict the minimum, 25th percentile, median, 75th percentile, and the maximum.

## Results

### Experiment 1

#### Passive membrane properties

Following the rupture of the patch membrane, the passive membrane properties of *C*_m_ and *V*_rest_ were measured as described in the “Materials and methods” section. Mean membrane capacitance for CTR, GUO and ADO groups are depicted in Fig. 1B. The capacitance values analyzed by a one-way ANOVA showed no significant differences among groups. Mean resting potential was measured in C-Clamp mode for the experimental groups and reported in Fig. 1C for CTR, GUO, and ADO groups. One-Way ANOVA did not outline significant differences between groups. The evaluation of *C*_m_ and *V*_rest_ in experiment 1 indicates that the treatments did not alter the passive membrane properties of SH-SY5Y cells. The *C*_m_ and *V*_rest_ values recorded were in accordance with those measured by other authors in the SH-SY5Y cell line within the same time range of plating and experimental conditions [26]. As previously reported [27–29], this cell line if adequately stimulated allows the study of sustained outward K^+^ currents. Representative recording traces, depicted in Fig. 1D, are elicited by 15 depolarizing steps in an extracellular solution containing TTX to eliminate the contribution of voltage-dependent Na^+^-channels, for CTR, GUO, and ADO experimental groups.

#### GUO increases outward K^+^ membrane currents in SH-SY5Y cells

**Fig. 2:**
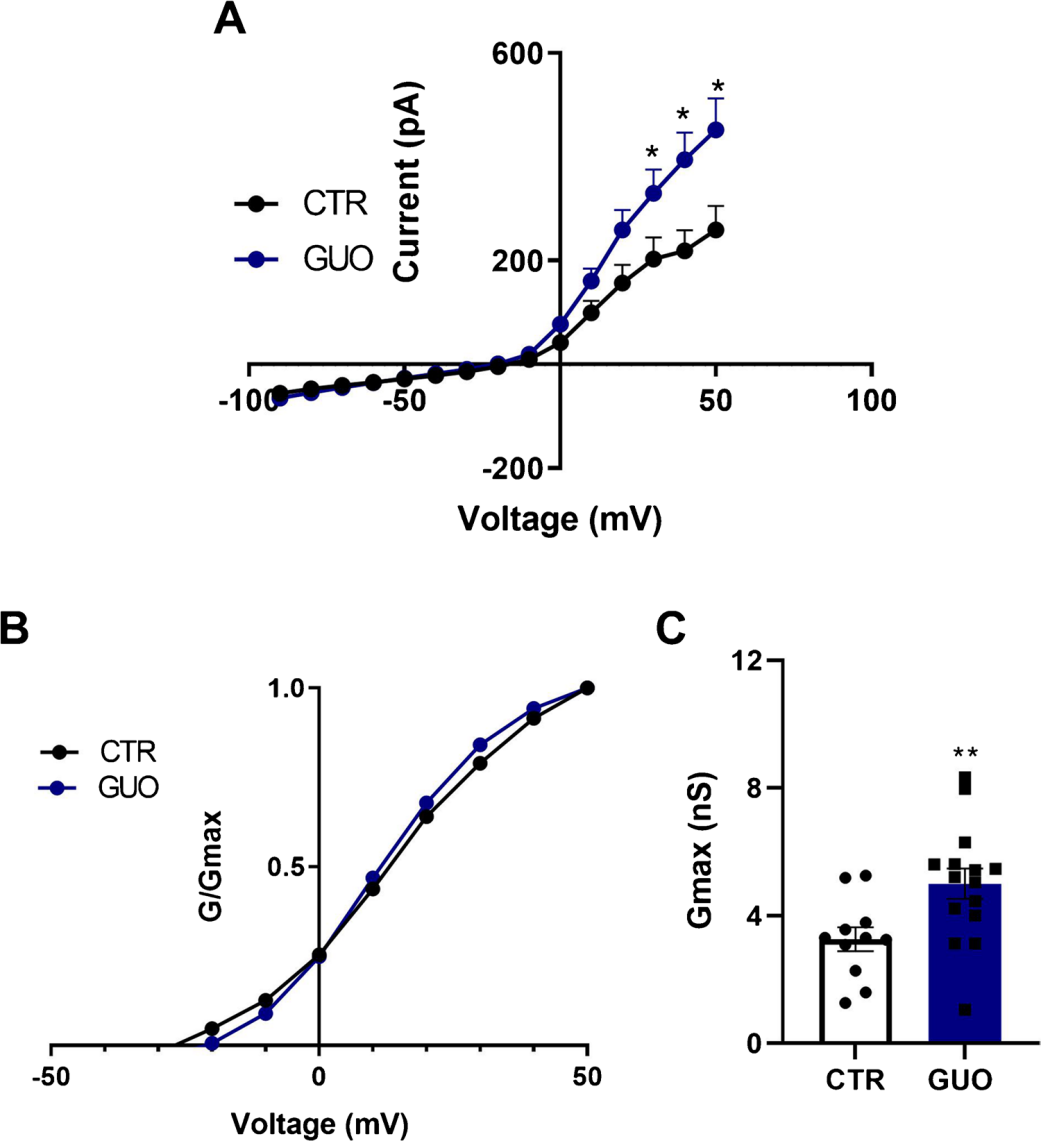
Voltage-dependent relationships of membrane currents in GUO-treated cells. **A** Current (pA)-voltage relationship (I-V) obtained in V-Clamp mode in SH-SY5Y cells. The I-V plot shows subtracted curves including cells in control (CTR) conditions and cells treated with guanosine (GUO), with TTX always added to the bath. Significant differences are indicated as (*) for *p* < 0.05 in GUO group vs CTR. **B** Comparison of K + channel voltage-dependence of activation curves. Curves show normalized K + conductances (*G*/*G*_max_) per voltage steps applied in GUO and CTR groups. **C** Maximal conductances for GUO and CTR groups calculated at + 50 mV. Statistical significance is indicated as (**) for *p* < 0.01 vs CTR

The outward current component, isolated when bath solution contained 1 µM TTX, showed a marked voltage-dependent sigmoidal activation, with similar activation and inactivation properties, as in [26], presumably constituting a delayed rectifier potassium current with a peak current at + 50 mV. To analyze the influence of GUO treatment on the active membrane currents, we evaluated I-V subtracted curves in subsequent depolarizing voltage steps from – 90 to + 50 mV (Fig. 2A) in GUO and CTR groups. A two-way ANOVA on GUO vs CTR revealed significant differences for voltage (F_(14, 195)_ = 57.74; *p* < 0.0001), treatment (F_(1, 195)_ = 22.93; *p* < 0.0001) and their interaction (F_(14, 195)_ = 3.38; *p* < 0.0001). In detail, post hoc analysis revealed that GUO significantly increased currents at + 30, + 40, and + 50 mV (*p* < 0.01).

Mean conductances were calculated at each test potential as described in the “Materials and methods” section with *E*_k_ derived by the Nerst equation. The conductances were normalized to *G*_max_ and plotted against the voltage applied (Fig. 2B). The curves, representing the fraction of activated K^+^ channels as a function of the membrane potential, show an increase in K^+^ conductance in GUO-treated cells. At potential eliciting the maximum current (i.e., + 50 mV), GUO increased *G*_max_ vs CTR as shown by unpaired *t*-test (*t* = 2.66, df = 24 and *p* = 0.0126; Fig. 2C). Even though the predominant role of K^+^ channels on outward currents in our experimental model has been ascertained by previous literature [29], we provided ultimate validation of a specific GUO action on outward K^+^ currents by administering GUO in a concomitant blockade of K^+^ channels with TEA. Remarkably, GUO + TEA treatment is able to significantly revert GUO-mediated potentiation on outward currents as evidenced by two-way ANOVA on voltage (F_(14, 270)_ = 63.50; *p* < 0.0001), treatment (F_(2, 270)_ = 3.24; *p* = 0.0405) and their interaction (F_(28, 270)_ = 7.36; *p* < 0.0001) in GUO + TEA compared to GUO and to baseline conditions (as shown in Supplementary Fig. 1).

#### Effects of GUO on membrane currents are assessed in relation to A1 and A2 receptors in SH-SY5Y cells

**Fig. 3:**
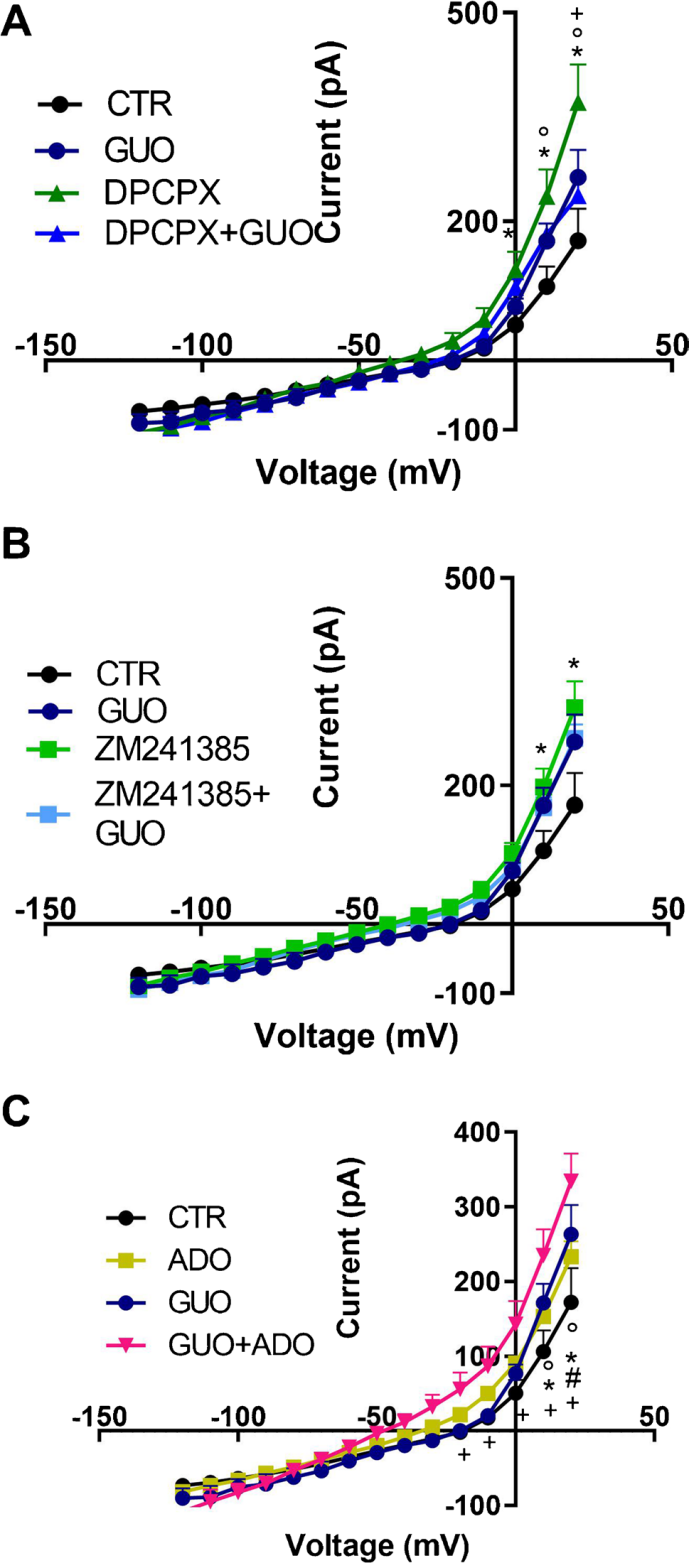
Voltage-dependent relationships of membrane currents in SH-SY5Y cells. **A** Current–voltage subtracted plot (I-V) obtained in V-clamp mode in control (CTR) conditions, in cells treated with guanosine (GUO) and A1 antagonist (DPCPX) and co-treated with both (DPCPX + GUO). Significant differences are indicated as (*) for *p* < 0.05 in DPCPX group vs CTR; as (+) for *p* < 0.05 in DPCPX + GUO group vs DPCPX; as (°) for *p* < 0.05 in GUO group vs CTR and vs DPCPX. **B** I-V subtracted plot obtained in V-Clamp mode in control (CTR) conditions, in cells treated with guanosine (GUO) and A2 antagonist (ZM241385) and co-treated with both (ZM241385 + GUO). Significant differences are indicated as (*) for *p* < 0.05 in ZM241385, GUO, and ZM241385 + GUO group vs CTR. **C** I-V subtracted plot obtained in V-clamp mode in control (CTR) conditions, in cells treated with guanosine (GUO) and adenosine (ADO) and co-treated with both (GUO + ADO). Significant differences are indicated as (*) for *p* < 0.05 in GUO vs CTR, as (°) for *p* < 0.05 in ADO vs CTR, as (#) for *p* < 0.05 in GUO + ADO vs CTR and ADO and as ( +) for *p* < 0.05 in GUO + ADO vs GUO

The effect of GUO on outward membrane currents in SH-SY5Y cells was studied in relation to specific ARs, A1 and A2, upon voltage-dependent stimulation (I-V subtracted curves in Fig. 3). The pretreatment with A1 antagonist (DPCPX) does not modify GUO-mediated increase in voltage-dependent K^+^ currents (see Fig. 3A).

A two-way ANOVA on GUO, DPCPX, DPCPX + GUO and CTR groups showed significant differences for voltage (F_(14, 405)_ = 156.0; *p* < 0.0001), pharmacological treatment (F_(3, 405)_ = 9.40; *p* < 0.0001) and their interaction (F_(42, 405)_ = 2.48; *p* < 0.0001). Interestingly, DPCPX significantly increases outward currents versus CTR at 0, + 10, and 20 mV and versus GUO at + 10 and 20 mV. The co-treatment DPCPX + GUO was not different vs GUO alone, but reduced membrane currents versus DPCPX at + 20 mV, thus suggesting that GUO is able to attenuate DPCPX potentiation of K^+^ membrane currents (Fig. 3A).

The pretreatment with A2 antagonist (ZM241385) does not modify the GUO-mediated increase in voltage-dependent K^+^ currents. A two-way ANOVA on GUO, ZM241385, ZM241385 + GUO and CTR groups was statistically significant for voltage (F_(14, 420)_ = 196.5; *p* < 0.0001), pharmacological treatment (F_(3, 420)_ = 9.08; *p* < 0.0001) and their interaction (F_(42, 420)_ = 1.5; *p* = 0.0268). Similarly to DPCPX alone, the post hoc Bonferroni test revealed that ZM241385 was able alone to increase outward membrane currents at + 10 mV and + 20 mV vs CTR, though not significantly different when co-treated with GUO.

Lastly, the influence of GUO in modulating outward currents in SH-SY5Y cells was assessed in presence of ADO, powerfully activating ARs. Statistical analysis performed by a two-way ANOVA comparing GUO, ADO and GUO + ADO co-treatment with CTR revealed significant differences between groups for voltage(F_(14, 405)_ = 181.3; *p* < 0.0001), pharmacological treatment (F_(3, 405)_ = 13.94; *p* < 0.0001) and their interaction (F_(42, 405)_ = 2.39; *p* < 0.0001). In detail, the post hoc Bonferroni test showed that ADO significantly increased voltage-dependent active outward currents at + 20 mV versus CTR and the co-treatment GUO + ADO significantly increased outward currents versus ADO, GUO, and CTR for *p* > 0.01 as in Fig. 3C.

#### ADO modulates outward membrane currents in relation to A1 and A2 receptors in SH-SY5Y cells

**Fig. 4:**
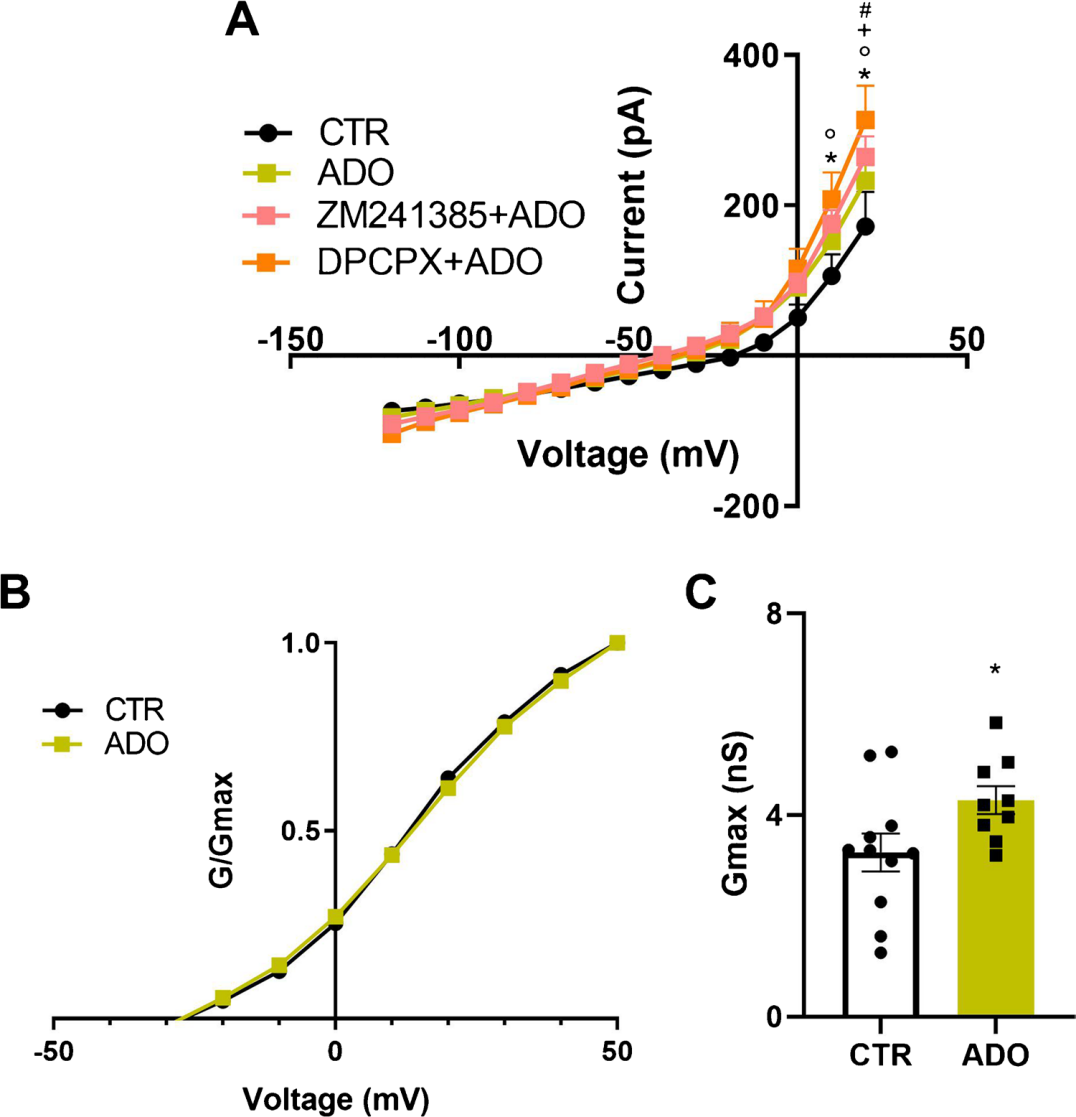
Voltage-dependent relationships of membrane currents in ADO-treated cells. **A** Current–voltage subtracted curves (I-V) obtained in V-clamp mode in SH-SY5Y cells. The I-V plot includes cells in control (CTR) conditions, cells treated with adenosine (ADO), cells treated with (DPCPX + ADO), and cells treated with (ZM241385 + ADO), with TTX always added to the bath. Significant differences are indicated as (*) for *p* < 0.05 in DPCPX + ADO group vs CTR and vs ADO, as (°) for *p* < 0.05 in ZM241385 + ADO group vs CTR, as (+) for *p* < 0.05 in DPCPX + ADO group vs ZM241385 + ADO and as (#) for *p* < 0.05 in ADO vs CTR group. **B** Comparison of K^+^ channel voltage dependence of activation curves. Curves show normalized K^+^ conductances (*G*/*G*_max_) per voltage steps applied in ADO and CTR groups. **C** Maximal conductances for ADO and CTR groups calculated at + 50 mV. Statistical significance is indicated as (*) for *p* < 0.05 vs CTR

The pharmacological competition on A1 and A2 receptors is assessed by administering ADO alone and pre-treated with DPCPX and ZM241385. A two-way ANOVA showed significant differences between groups for voltage (F_(14, 435)_ = 198.6; *p* < 0.0001), pharmacological treatment (F(_3, 435)_ = 7.63; *p* < 0.0001) and their interaction (F_(42, 435)_ = 2.391.87; *p* = 0.0011). In particular, both co-treatments, DPCPX + ADO and ZM241385 + ADO, increased the membrane currents versus CTR and also DPCPX + ADO potentiated outward currents vs ADO alone, as in Fig. 4A. Mean conductances for ADO were calculated as in “Materials and methods” section, normalized to *G*_max_ and plotted against voltage applied (Fig. 4B). Following ADO treatment, the mean values of *G*_max_ at potential eliciting the maximum current (i.e., + 50 mV), induced an increase in K^+^ conductances versus CTR as shown by unpaired *t*-test (*t* = 2.12, df = 18, and *p* = 0.048; Fig. 4C).

### Experiment 2

#### GUO and ADO both modify outward membrane currents after 24 h treatment and influence AP

**Fig. 5:**
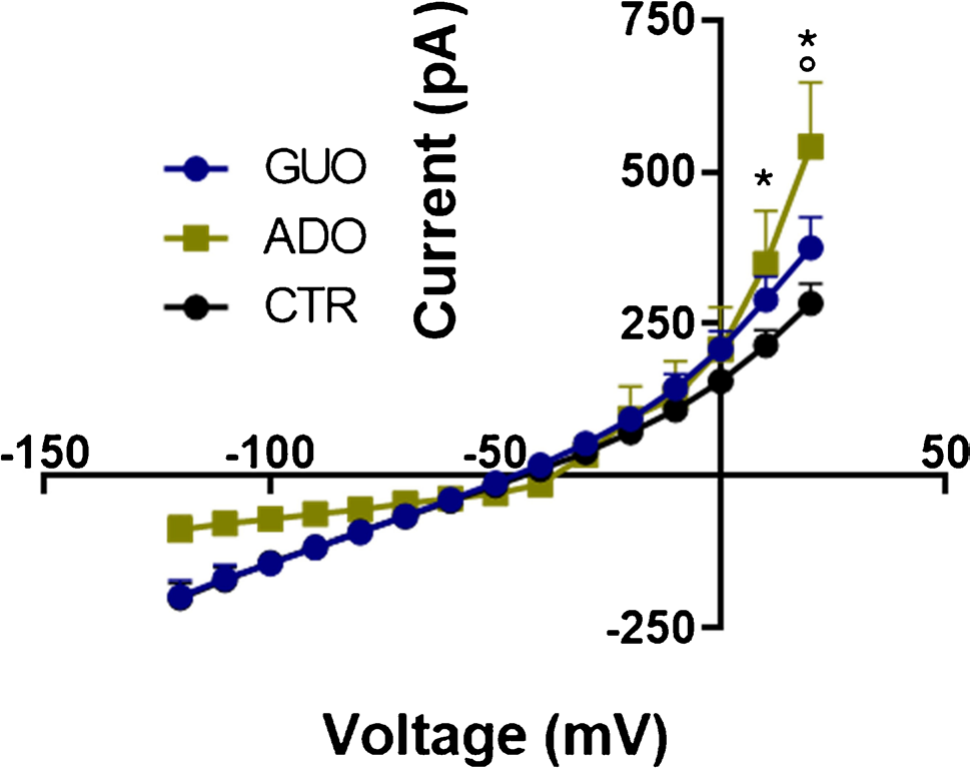
Voltage-dependent relationships of membrane currents in SH-SY5Y cells treated for 24 h with guanosine (GUO) and adenosine (ADO). Current–voltage plot (I-V) obtained in V-clamp mode in CTR, GUO, and ADO groups. Significant differences are indicated as (*) for *p* < 0.05 in ADO vs CTR, as (°) for *p* < 0.05 in GUO vs ADO and vs CTR

In experiment 2, we assessed the effects of GUO and ADO following a 24-h treatment on outward membrane currents isolated when bath solution contained 1 microM TTX. Statistical analyses by a two-way ANOVA proved significant differences for voltage (F_(14, 495)_ = 95.2; *p* < 0.0001), pharmacological treatment (F_(2, 495)_ = 12.28; *p* < 0.0001) and their interaction (F_(28, 495)_ = 1.59; *p* = 0.028) between groups. In detail, the post hoc test showed a significant increase in membrane current in GUO and ADO groups respectively vs CTR as in Fig. 5. Also, ADO significantly increased outward currents with respect to GUO at + 20 mV (*p* < 0.01).

Furthermore, the effect of GUO and ADO on the ability of undifferentiated SH-SY5Y cells to generate a single AP was analyzed in response to 15 rectangular current injections of 10 pA for 500 ms starting from a hyperpolarized holding potential of approximately − 90 mV [26].

**Fig. 6:**
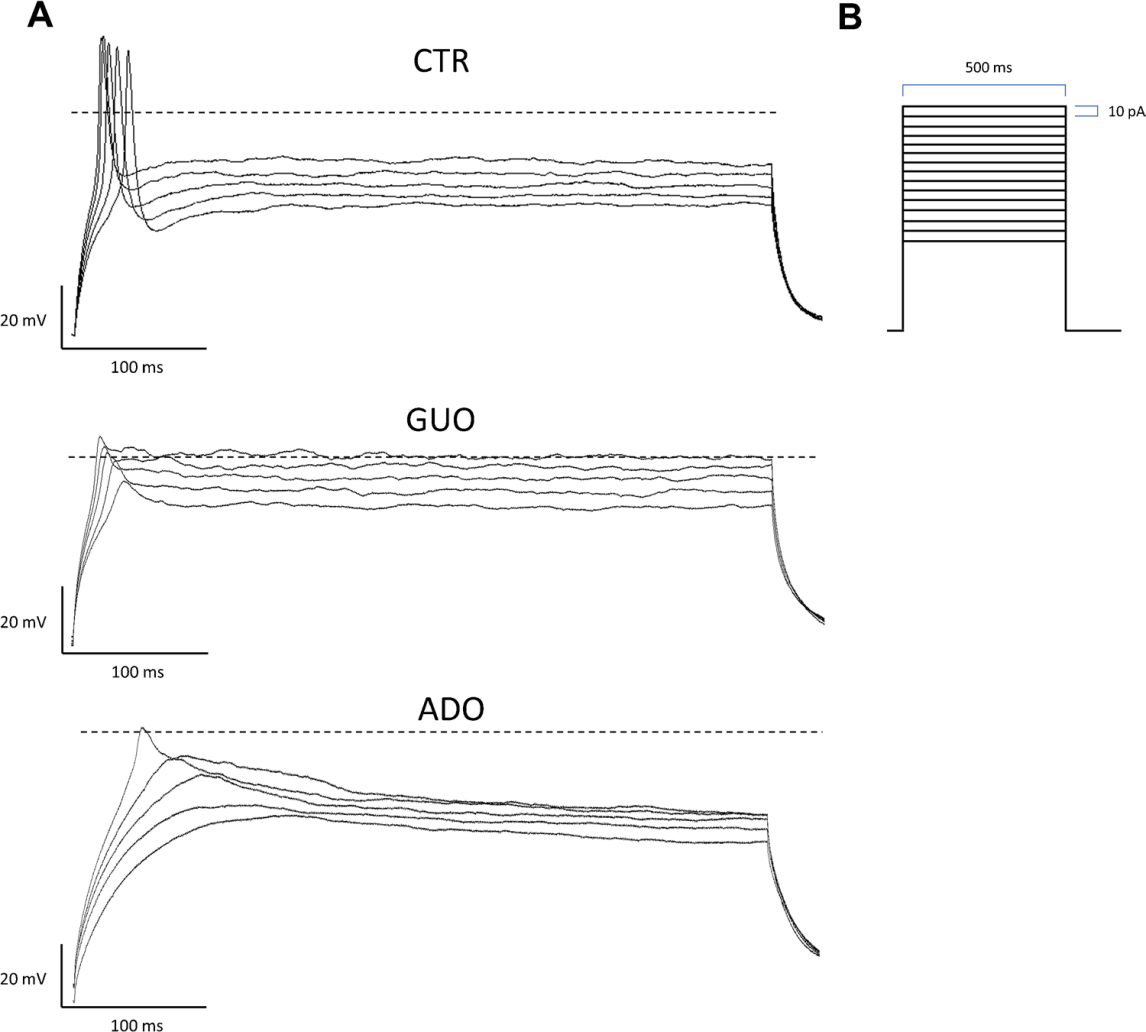
Sample whole-cell recordings of action potentials in SH-SY5Y cells in C-clamp mode. Representative traces showing action potentials recorded in CTR, GUO, and ADO groups (**A**) in response to 15 subsequent depolarizing current pulses of 500-ms duration in 10-pA steps (**B**)

Only some cells per group in experimental conditions described in the “Materials and methods” section, namely devoid of TTX in the extracellular culture medium, were able to generate overshoot AP (see Table 1 and representative traces in Fig. 6), the others failed to generate an AP or presented abortive AP. In particular, we obtained 85% of CTR cells (i.e., 17 out of 20 recorded cells) that fired AP, whereas upon GUO and ADO treatments this percentage of AP was reduced (GUO group: 39.58%, i.e., 19 responding cells out of 48; and ADO group: 16.6%, i.e., 3 out of 18). The cells able to emit AP were further analyzed to explore whether drug treatments were able to modify the AP parameters.

**Table 1:** Electrophysiological properties of action potentials in SH-SY5Y cells treated for 24 h with GUO and ADO

	**CTR**	**GUO**	**ADO**
AP amplitude (mV)	95.08 ± 23.41	84.01 ± 13.49	44.87 ± 22.99*
Depolarization time (ms)	3.94 ± 1.74	4.80 ± 1.5	5.76 ± 1.16
Repolarization time (ms)	5.95 ± 4.39	1.20 ± 1.94*	0.04 ± 0.11*
Event frequency (Hz)	0.42 ± 0.18	0.18 ± 0.27*	0.02 ± 0.07*

Data represent the mean ± SD obtained only in cells presenting overshoot AP. The amplitude was determined as the voltage difference between the *V*_rest_ and the peak; the time to reach a positive peak represents the “depolarization time,” ms; and the time to repolarize and reach the negative peak (“repolarization time,” ms)

Significant differences are indicated for *p* < 0.05 (*) vs CTR

As for the cumulative mean peak amplitude of action potentials, one-way ANOVA revealed significant differences between groups (F_(3,38)_ = 8.99; *p* = 0.0007), particularly ADO was able to reduce mean AP amplitude with respect to CTR (*p* < 0.01) and to GUO (*p* < 0.05; Table 1). Moreover, we evaluated the influence of GUO and ADO on the event frequency. One-way ANOVA revealed that both treatments statistically reduce the frequency (F_(2, 84)_ = 14.02; *p* < 0.0001, Table 1) of AP when compared to controls. Finally, the effect of GUO and/or ADO in the AP depolarization/repolarization time was assessed. One-way ANOVA showed that GUO and ADO treatments did not alter the depolarization time when compared with CTR cells. However, when we analyzed the repolarization time of AP, significant differences (F_(2,38)_ = 11.21; *p* < 0.0001) were found. Indeed, post hoc analysis revealed that GUO and ADO respectively reduce repolarization time vs CTR (p < 0.05, Table 1).

## Discussion

Homeostatic regulation of cellular excitability is achieved through a variety of ion channels, including K^+^ channels [36], and plays a key role in neuroprotection against certain pathological conditions [37, 38]. Within this framework, GUO could exert a potential role in cellular excitability that has not been fully explored yet. Thus, we investigated the modulatory effects of GUO on the bioelectric properties of human neuroblastoma SH-SY5Y cells. These cells are widely used both for their easy culturing conditions and for the feasibility to study discrete biophysical properties, including K^+^ currents [26, 29, 39, 40]. Moreover, multiple effects of GUO were described in this cell line, including neuroprotection and differentiation [22, 25], some of which were specifically mediated by K^+^ currents.

When targeting the electrophysiological properties of these cell lines, it is crucial that these cells have voltage-gated channels in the plasma membrane [41, 42] that drive their ability to emit AP, usually a single AP in undifferentiated or even bursts in differentiated ones [29], similarly to neurons [43]. Even more importantly, the opening and closure of these voltage-gated channels are mainly responsible for inward and outward membrane currents recorded in V-clamp mode, as demonstrated by the application of selective blockers of voltage-gated channels, i.e., TTX and. In particular, inward currents are predominantly Na^+^-dependent and TTX-sensitive [28], whereas outward currents, being inactivated by TEA, cesium (Cs), and 4-aminopyridine, are largely produced by delayed rectifying K^+^ currents [26, 29, 44], though inward Na^+^ currents show much less driving force with respect to K^+^ outward currents [27], probably due to a reduced population of voltage-dependent Na^+^ channels present in these cells. These well-known electrophysiological features of SH-SY5Y cells, together with their molecular properties, proved this experimental model is particularly suitable for focusing on K^+^-mediated processes, in specific recording conditions.

When GUO is applied to these cells, passive membrane properties seemed unchanged in our experimental conditions, while we revealed modifications in the active electrical membrane properties. The main outcomes obtained are that GUO managed to increase the cumulative amplitude of outward membrane currents, specifically modulating mean K^+^ conductances and the relative activation curve, all considered a function of voltage-dependent steps. Other authors have previously reported an inhibitory effect of chronic (6 days) GUO treatment on Na^+^ currents, due to the reduction in sodium channel expression [45]. Besides, indirect pharmacological evidence has described a putative involvement of calcium-activated potassium channels in specific GUO neuroprotective effects [20–22]. In agreement, our results support a possible role of GUO on cellular excitability, putatively modulating the hyperpolarized state of cell membranes that would exert inhibitory activity.

Considering the still unveiled role of GUO in purinergic molecular transmission and the already described involvement of ARs in GUO effects, we aimed to investigate if the GUO-dependent effects on K^+^ currents in SH SY5Y cells could implicate the activity of the ARs. Thus, we administered two widely used antagonists/inverse agonists of A1 and A2 subtypes (i.e., DPCPX and ZM241385 [46]) alone and in combination with GUO. When we targeted A1 and A2 receptors, the administration of DPCPX and ZM241385 alone induced an increase in the active voltage-dependent K^+^ currents. Noticeably, only the pretreatment with A1R inverse agonist to GUO-administered cells was able to reduce the effects of A1R alone, hinting that GUO can attenuate the potentiation exerted by DPCPX. This piece of evidence suggests a possible implication of GUO in the effects mediated by A1Rs. Various possible explanations could hint that GUO owns an allosteric binding site on A1R and/or GUO itself activates its specific orphan receptor that in turn modulates A1 activity.

Then, to better focus on the role of ARs in GUO-mediated effects, we administered ADO alone, in combination with GUO. Our data proved that ADO alone could augment K^+^ outward currents and the activity of ADO is modified by the competition with the specific inverse agonists of ARs, especially with subtype A1R. Previous investigations already demonstrated ADO-mediated activation of K^+^ currents in neuronal cells [47–50], especially on G-protein activated inward rectifying K^+^ (GIRK) currents, though these channels have not been discovered in undifferentiated SH-SY5Y cells so far [51, 52].

Noteworthy, the concurrent GUO and ADO administration is able to potentiate synergistically the effects of both nucleosides on delayed rectifying K^+^ currents. These novel results could shed more light on the interlink between GUO and ADO on cellular excitability, hypothesizing an additive activity focused on K^+^ currents or a co-activation directed on ARs.

A further purpose emerging from our investigation was to assess whether a 24-h treatment with GUO could affect the electrophysiological properties of SH-SY5Y cells as well as acute administration. Our findings from experiment 2 not only revealed that GUO is still able to increase the K^+^-dependent outward membrane currents, but also ADO improves them to a greater extent, probably because in a semi-chronic fashion multiple secondary effects are triggered such as numerous cellular signal cascades and transcriptional regulation.

In addition, SH-SY5Y cells were tested in a C-clamp configuration to observe eventual modifications to AP emitted upon rectangular current injections in a medium containing GUO or ADO for 24 h. As previously reported [26], only a defined percentage of SH-SY5Y cells is able to generate AP and this can be influenced by drug treatments, since GUO and ADO reduced the percentage of cells firing AP. In the number of cells still able to generate AP, we also evaluated the AP electrophysiological parameters that eventually changed following GUO and ADO administration. Crucially, both nucleosides affected the repolarization time by decreasing its duration, without affecting the depolarization time, thereby supporting a specific modulation of K^+^-outward currents.

In this context, GUO effects could be performed via an independent action exerted through its orphan membrane receptors and/or via the on-demand recruitment of ARs. Indeed, GUO could be implicated in the process of activation of ARs, especially the A1 subtype, ultimately leading to allosteric modulation of ARs alone or via a receptorial complex with orphan GUO receptors.

In conclusion, we described here for the first time a GUO-mediated regulation of cellular excitability via enhancement of K^+^ outward currents in SH-SY5Y cells, with a putative functional interaction with adenosinergic signaling. Further research should be carried out to provide a detailed characterization of the molecular mechanisms behind GUO-mediated modulation of K^+^ channel gating and/or biogenesis in native conditions. Overall, the discovery of this GUO role in the bioelectrical activity of SH-SY5Y cells might open brand-new scenarios further exploring nucleosides in neuronal excitability and related disorders.

## Supplementary Information

Below is the link to the electronic supplementary material.

Supplementary file1 (DOCX 12 KB)

Supplementary file2 (JPG 57 KB)

## Data Availability

The data that support the findings of this study are available on reasonable request.
