# Supplementary material for: Guanosine modulates K+ membrane currents in SH-SY5Y cells: involvement of adenosine receptors
Source: Pflugers Arch. 2022 Sep 1;474(11):1133–45. doi: 10.1007/s00424-022-02741-4 (PMC9560947; doi:10.1007/s00424-022-02741-4)
Supplement: Supplementary file 1 — Supplementary file1 (DOCX 12 KB) [file 424_2022_2741_MOESM1_ESM.docx]

**Supplementary Figure Legend** Voltage-dependent relationships of membrane currents in GUO-treated cells alone and in combination with TEA. The I-V plot shows subtracted curves including cells in control (CTR) conditions and cells treated with guanosine (GUO) alone and in combination with TEA. Significant differences are indicated for p<0.05 as (*) in GUO group vs CTR, as (°) for GUO vs GUO+TEA and as (+) for GUO+TEA vs CTR.
