# Supplementary figures and images for: Guanosine modulates K+ membrane currents in SH-SY5Y cells: involvement of adenosine receptors
Source: Pflugers Arch. 2022 Sep 1;474(11):1133–45. doi: 10.1007/s00424-022-02741-4 (PMC9560947; doi:10.1007/s00424-022-02741-4)

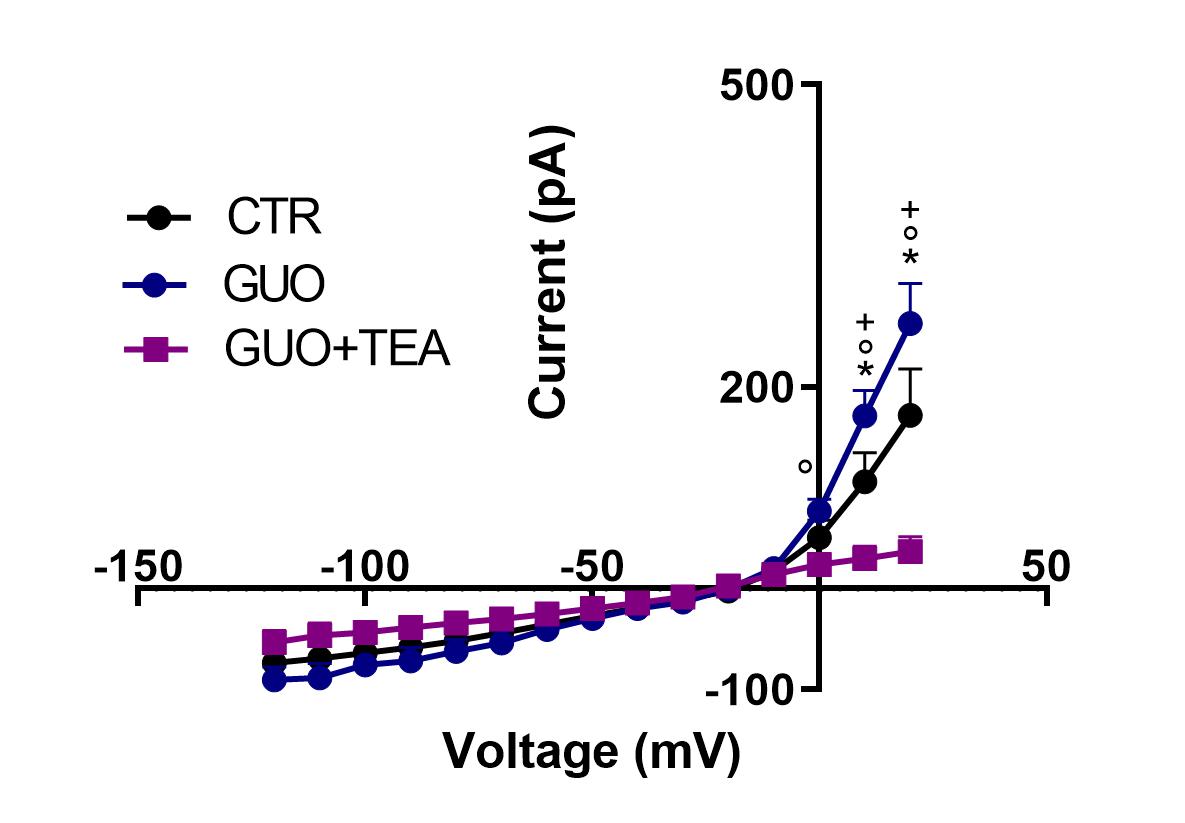

Supplement: Supplementary file 2 — Supplementary file2 (JPG 57 KB) [file 424_2022_2741_MOESM2_ESM.jpg]
